# The prevalence and nature of multi‐type child maltreatment in Australia

**DOI:** 10.5694/mja2.51868

**Published:** 2023-04-02

**Authors:** Daryl J Higgins, Ben Mathews, Rosana Pacella, James G Scott, David Finkelhor, Franziska Meinck, Holly E Erskine, Hannah J Thomas, David M Lawrence, Divna M Haslam, Eva Malacova, Michael P Dunne

**Affiliations:** ^1^ Institute of Child Protection Studies Australian Catholic University Melbourne VIC; ^2^ Queensland University of Technology Brisbane QLD; ^3^ Bloomberg School of Public Health Johns Hopkins University Baltimore MD United States of America; ^4^ Institute for Lifecourse Development University of Greenwich London United Kingdom; ^5^ The University of Queensland Brisbane QLD; ^6^ QIMR Berghofer Medical Research Institute Brisbane QLD; ^7^ Crimes against Children Research Center University of New Hampshire Durham NH United States of America; ^8^ University of Edinburgh Edinburgh United Kingdom; ^9^ North‐West University Potchefstroom South Africa; ^10^ Queensland Centre for Mental Health Research Brisbane QLD; ^11^ Curtin University Perth WA; ^12^ Institute for Community Health Research Hue University Hue City Vietnam

**Keywords:** Child abuse, Child health, Mental disorders, Epidemiology

## Abstract

**Objectives:**

To determine the prevalence in Australia of multi‐type child maltreatment, defined as two or more maltreatment types (physical abuse, sexual abuse, emotional abuse, neglect, or exposure to domestic violence) and to examine its nature, family risk factors, and gender and age cohort differences.

**Design:**

Retrospective cross‐sectional survey using a validated questionnaire.

**Setting and participants:**

Mobile phone random digit‐dial sample of the Australian population aged 16 years and older.

**Main outcome measures:**

National estimates of multi‐type child maltreatment up to age 18 years using the Juvenile Victimisation Questionnaire‐R2: Adapted Version (Australian Child Maltreatment Study).

**Results:**

Of 8503 participants, 62.2% (95% CI, 60.9–63.6%) experienced one or more types of child maltreatment. Prevalence of single‐type maltreatment was 22.8% (95% CI, 21.7–24.0%), whereas 39.4% (95% CI, 38.1–40.7%) of participants reported multi‐type maltreatment and 3.5% (95% CI, 3.0–4.0%) reported all five types. Multi‐type maltreatment was more common for gender diverse participants (66.1% [95% CI, 53.7–78.7%]) and women (43.2% [95% CI, 41.3–45.1%]) than for men (34.9% [95% CI, 33.0–36.7%]). Multi‐type maltreatment prevalence was highest for those aged 25–44 years. Family‐related adverse childhood experiences — especially mental illness and alcohol or substance misuse — increased risk. Exposure to domestic violence was the maltreatment type most often present in multi‐type maltreatment patterns.

**Conclusions:**

Multi‐type child maltreatment is prevalent in Australia and more common in women and gender diverse individuals. Child protection services, health practitioners, and prevention and intervention services must assess and manage multi‐type maltreatment in children and address its health consequences across the lifespan. Public health policy should consider prevention services or strategies that target multi‐type child maltreatment.

**The known:** Although we know that child maltreatment is common, we know little about the prevalence of exposure to multiple forms of maltreatment (physical abuse, emotional abuse, sexual abuse, neglect, and exposure to domestic violence).

**The new:** To our knowledge, this is the first time that a population‐representative study of all five child maltreatment types has been conducted. Australian children experienced multi‐type maltreatment more often than a single type (39.4% *v* 22.8%). Almost one‐quarter (23.3%) experienced three to five maltreatment types, and 3.5% experienced all five types. A common multi‐type maltreatment combination involves exposure to domestic violence, emotional abuse and physical abuse. Broader family‐related adverse experiences almost doubled the risk of multi‐type maltreatment.

**The implications:** Prevention, protection and treatment services must coordinate to promote safety and recovery from multi‐type maltreatment. Public health prevention measures must employ broad strategies addressing multi‐type maltreatment, particularly targeting women and gender diverse individuals.

Different forms of child maltreatment — physical abuse, sexual abuse, emotional abuse, neglect, and exposure to domestic violence — are associated with substantial adverse effects throughout life on mental health, physical health and health risk behaviour.^1^, ^2^ However, most research considers bilateral relationships between an individual maltreatment type and measures of wellbeing. Unless the totality of a person's experience of different types of maltreatment is measured, researchers and clinicians may misattribute outcomes to one type of maltreatment. Further, outcomes attributed to individual maltreatment types cannot simply be added to understand the consequences of multiple forms of maltreatment.

This insight, together with clinical understanding of victim‐survivors’ lived experience, underpinned the conceptualisation of exposure to multiple forms of child maltreatment and its consequent harms as “multi‐type maltreatment”.^3^, ^4^ Some studies suggest that multi‐type maltreatment is common.^5^, ^6^ One of the first comprehensive analyses of multiple forms of child maltreatment in the United States examined it as a subset of other childhood victimisation experiences such as bullying and community violence.^7^ In a convenience sample examining four types of child maltreatment in 2292 children (aged 5–13 years), 23.9% reported two to four maltreatment types.^8^ In a random sample of children in the US, 56.8% of those who witnessed family violence experienced another type of maltreatment, most commonly psychological abuse (38.2%) or physical abuse (31.1%).^9^ Similar patterns have been found in quasi‐randomised youth samples in Vietnam and Malaysia.^10^, ^11^ A recent systematic review of research on child maltreatment in China emphasised the predominance of studies on single or few types of maltreatment, and the paucity of research on multi‐type maltreatment.^12^

In Australian research conducted using a non‐representative community sample, half of participants who experienced any type of maltreatment also reported at least one other type.^3^ A recent study pooled various data sources in Australia to estimate the proportion of maltreated individuals where there was co‐occurrence (looking at four types of maltreatment, excluding exposure to domestic violence). They found very high proportions of co‐occurrence, ranging from 57.1% for sexual abuse to 91.0% for emotional abuse, indicating that multi‐type maltreatment is the more typical experience of child maltreatment than single type.^13^ A meta‐analysis of co‐occurrence rates of family victimisation found significantly higher rates among the clinical population (36.0%) than the general population (9.7%).^14^ Although recent studies have assessed health outcomes such as pre‐pregnancy obesity and found them significantly related to various types of child maltreatment, the risk for those who have experienced multi‐type maltreatment is not known.^15^

Despite its importance for policy and clinical practice, longstanding gaps in the international evidence base persist.^5^, ^6^ Our understanding of the prevalence and nature of multi‐type maltreatment at the population level and the associated health outcomes is limited.^16^, ^17^ A recent systematic review identified only one study that considered all five types of maltreatment, but the study used a non‐representative sample of 358 children.^6^, ^18^ This review also found pronounced gaps in evidence relating to multi‐type maltreatment involving emotional abuse and exposure to domestic violence.^6^ To our knowledge, no study to date has ascertained the prevalence, nature and associated family‐related risk factors for multi‐type child maltreatment (up to age 18 years) of all five forms in a population‐representative sample.

The Australian Child Maltreatment Study (ACMS) conducted a national survey of a random sample of the population aged 16 years and older, and found that each form of maltreatment is common. Prevalence rates were: neglect, 8.9%; sexual abuse, 28.5%; emotional abuse, 30.9%; physical abuse, 32.0%; and exposure to domestic violence, 39.6%.^19^, ^20^ In this article, we build on those findings, with the aims of establishing the first source of evidence on the prevalence in Australia of any multi‐type maltreatment and different multi‐type maltreatment combinations, and identifying gender and age‐group differences. Accordingly, we examine three research questions:

▪ What is the prevalence of multi‐type maltreatment?
▪ What is the prevalence of experiencing different combinations of maltreatment domains?
▪ What family‐related adverse childhood experiences are associated with great risk of single‐type and multi‐type maltreatment?

## Method

### Participants

As detailed elsewhere in this supplement, we recruited a representative sample of Australians aged 16 years and older by random digit‐dial via an advance text message inviting participation, with a follow‐up phone call.^21^ We asked participants to describe their gender. Interviewers were able to code responses against 13 categories or transcribe verbatim any other response. As well as using data for women and men, we collapsed all other responses into the category of diverse genders.

### Outcome measures

We administered the Juvenile Victimisation Questionnaire‐R2: Adapted Version (Australian Child Maltreatment Study). The 16 screener items measured all five types of child maltreatment up to age 18 years, as defined in the ACMS protocol and further explained elsewhere in this supplement.^19^, ^22^ The questionnaire also included questions about other adverse childhood experiences, including four family‐related risk factors: parental separation or divorce; living with someone who was mentally ill, suicidal or severely depressed; living with someone who had a problem with alcohol or drugs; and family economic hardship.^21^

We selected these risk factors for analysis for several reasons. First, each is common enough to provide usable data, compared with others such as parental incarceration. Second, they are supported in the literature as associated with individual maltreatment types in a more robust manner than other adverse childhood experiences, and we deemed it important to assess their association with multi‐type maltreatment in this analysis. Third, they are significant scientifically and relevant for policy because they are more readily modifiable than some other adverse childhood experiences. Fourth, the Adverse Childhood Experiences Scale is acknowledged as not including all relevant adversities,^22^, ^23^ so assessment of all its standard items would be subject to limitations.

### Statistical analysis

We calculated survey‐weighted prevalence (with 95% confidence intervals) of physical abuse, sexual abuse and exposure to domestic violence, based on positive endorsement of any of the screener items for these maltreatment types, regardless of how many times the experience happened. For emotional abuse and neglect, we calculated prevalence only if the experience occurred over a period of weeks, months or years.^20^

We defined multi‐type maltreatment as the experience of two or more of the five child maltreatment types across childhood and adolescence. There are 26 potential multi‐type maltreatment combinations: experiences of two types (ten combinations), three types (ten combinations), four types (five combinations) or all five types. We split the sample into three mutually exclusive groups: no maltreatment, single‐type maltreatment, and multi‐type maltreatment. The multi‐type maltreatment group was further divided into the number of maltreatment types experienced (two, three, four or five). We also made comparisons by age group: the youngest cohort (participants aged 16–24 years), the middle cohort (collapsed data for participants aged 25–34 and 35–44 years), and the oldest cohort (collapsed data for participants aged 45–54, 55–64 and ≥ 65 years).

To measure associations between family‐related adverse childhood experiences and multi‐type maltreatment, we considered participants’ experiences of the four selected family‐related risk factors. For each of these, we calculated the relative risk (RR) and 95% confidence interval, comparing each maltreatment grouping with all others: no maltreatment was compared with one type and with two or more types; one type was compared with no maltreatment and with two or more types; and two or more types was compared with no maltreatment and with one type. We calculated RRs using log binomial regression, accounting for the survey weights. We did not consider the contribution of potential confounders as it was beyond the scope of the study and would have required a separate detailed analysis. Our primary goal was to consider the presence of these risk factors in the context of multi‐type maltreatment, which involved a novel analysis of the interplay between a substantial number of combinations of maltreatment types and family‐related adverse childhood experiences.

All analyses were conducted using SAS 9.4 or Stata 17.0. Two of us (DMH and DL) randomly spot‐checked the SAS coding and results in SPSS 27.

### Ethics approval

The Queensland University of Technology Human Research Ethics Committee approved the study (1900000477). Participants gave informed consent.

## Results

### Prevalence of multi‐type maltreatment

In total, 8503 participants completed the survey; 3503 were aged 16–24 years, 2000 were aged 25–44 years) and 3000 were aged ≥ 45 years. In this sample, 5280 participants (62.2% [95% CI, 60.9–63.6%]) reported experiencing one or more types of child maltreatment, 3378 participants (39.4% [95% CI, 38.1–40.7%]) reported experiencing any multi‐type child maltreatment (ie, 2–5 types), and 286 participants (3.5% [95% CI, 3.0–4.0%]) reported experiencing all five types. Experiencing three types was reported by 1056 participants (11.7% [95% CI, 10.8–12.6%]) and experiencing four types was reported by 694 participants (8.1% [95% CI, 7.4–8.8%]). This meant that 2036 participants (23.3% [95% CI, 22.1–24.4%]) experienced three to five types of child maltreatment and 980 participants (11.6% [95% CI, 10.7–12.4%]) experienced four to five types (Box 1).

Box 1 **Prevalence of multi‐type maltreatment by number of maltreatment types, and by gender and age cohort (*N* = 8503)**

**Table:** 

	Participants — number; percentage (95% CI)						
	No maltreatment	One type of maltreatment	Any multi‐type maltreatment (≥ 2 types)	Two types of maltreatment	Three types of maltreatment	Four types of maltreatment	Five types of maltreatment
**All ages**	3223; 37.8% (36.4–39.1%)	1902; 22.8% (21.7–24.0%)	3378; 39.4% (38.1–40.7%)	1342; 16.1% (15.1–17.1%)	1056; 11.7% (10.8–12.6%)	694; 8.1% (7.4–8.8%)	286; 3.5% (3.0–4.0%)
Women	1402; 34.5% (32.6–36.3%)	928; 22.4% (20.7–24.0%)	1852; 43.2% (41.3–45.1%)	668; 15.6% (14.2–17.0%)	567; 12.7% (11.4–13.9%)	421; 10.2% (9.1–11.4%)	196; 4.7% (3.9–5.5%)
Men	1804; 41.6% (39.7–43.5%)	954; 23.5% (21.8–25.1%)	1437; 34.9% (33.0–36.7%)	656; 16.7% (15.2–18.2%)	461; 10.5% (9.4–11.7%)	243; 5.6% (4.7–6.4%)	77; 2.0% (1.5–2.6%)
Diverse genders	17; 18.5% (7.2–29.7%)	20; 15.4% (6.7–24.1%)	89; 66.1% (53.7–78.7%)	18; 15.0% (5.3–24.6%)	28; 17.7% (9.0–26.4%)	30; 21.1% (11.4–30.7%)	13; 12.4% (3.6–21.2%)
**16–24 years**	1368; 38.8% (37.0–40.6%)	732; 21.0% (19.5–22.5%)	1400; 40.2% (38.4–42.0%)	534; 14.8% (13.5–16.1%)	454; 13.1% (11.8–14.4%)	285; 8.6% (7.5–9.6%)	127; 3.7% (3.0–4.4%)
Women	565; 34.5% (31.9–37.0%)	340; 19.9% (17.8–22.1%)	757; 45.6% (42.9–48.3%)	267; 15.4% (13.5–17.3%)	237; 14.0% (12.2–15.9%)	163; 10.5% (8.9–12.2%)	90; 5.6% (4.4–6.8%)
Men	794; 44.5% (41.9–47.1%)	397; 22.4% (20.2–24.7%)	575; 33.0% (30.6–35.5%)	254; 14.4% (12.6–16.2%)	194; 11.5% (9.8–13.2%)	99; 5.7% (4.5–6.9%)	28; 1.4% (0.9–2.0%)
**25–44 years**	682; 33.4% (31.2–35.7%)	464; 22.5% (20.5–24.5%)	854; 44.0% (41.6–46.4%)	352; 18.3% (16.4–20.2%)	245; 12.3% (10.7–13.8%)	178; 9.0% (7.6–10.4%)	79; 4.4% (3.4–5.5%)
Women	294; 29.6% (26.5–32.8%)	223; 21.2% (18.4–24.0%)	469; 49.2% (45.7–52.6%)	170; 17.6% (14.9–20.2%)	130; 13.1% (10.8–15.4%)	117; 12.4% (10.1–14.7%)	52; 6.2% (4.4–7.9%)
Men	385; 37.5% (34.2–40.9%)	237; 24.1% (21.1–27.1%)	370; 38.4% (35.0–41.7%)	179; 19.2% (16.4–22.0%)	110; 11.3% (9.0–13.5%)	57; 5.5% (4.0–7.0%)	24; 2.4% (1.4–3.5%)
**≥ 45 years**	1173; 40.5% (38.5–42.4%)	706; 23.5% (21.8–25.2%)	1124; 36.0% (34.1–37.9%)	456; 14.9% (13.5–16.3%)	357; 11.0% (9.7–12.2%)	231; 7.3% (6.3–8.4%)	80; 2.8% (2.1–3.4%)
Women	543; 37.6% (34.8–40.3%)	365; 23.7% (21.3–26.1%)	626; 38.7% (36.0–41.5%)	231; 14.3% (12.4–16.2%)	200; 12.1% (10.3–13.9%)	141; 8.8% (7.2–10.4%)	54; 3.5% (2.5–4.5%)
Men	625; 43.8% (41.0–46.7%)	338; 23.4% (20.9–25.8%)	492; 32.8% (30.1–35.5%)	223; 15.5% (13.4–17.6%)	157; 9.8% (8.1–11.4%)	87; 5.6% (4.3–6.9%)	25; 1.9% (1.1–2.7%)

More than one‐third of participants (3223; 37.8% [95% CI, 36.4–39.1%]) reported no maltreatment. A smaller proportion (1902; 22.8% [95% CI, 21.7–24.0%]) reported only one type of maltreatment, and the highest rates of single‐type maltreatment were for exposure to domestic violence (755; 8.4% [95% CI, 7.6–9.1%]) and sexual abuse (518; 6.7% [95% CI, 6.0–7.4%]) (Supporting Information, table 1).

Considering gender differences, the rate of any multi‐type maltreatment was substantially higher for women (1852; 43.2% [95% CI, 41.3–45.1%]) than for men (1437; 34.9% [95% CI, 33.0–36.7%]) and was highest for participants with diverse genders (89; 66.1% [95% CI, 53.7–78.7%]). Women experienced higher prevalence of four and five types of multi‐type maltreatment compared with men (eg, five types: 196; 4.7% [95% CI, 3.9–5.5%] *v* 77; 2.0% [95% CI, 1.5–2.6%]), and these rates were higher still for participants identifying with diverse genders.

Considering age group differences, the middle cohort (25–44 years) had the highest prevalence of any multi‐type maltreatment (854; 44.0% [95% CI, 41.6–46.4%]), followed by the youngest cohort (16–24 years) (1400; 40.2% [95% CI, 38.4–42.0%]) and then the oldest cohort (≥ 45 years) (1124; 36.0% [95% CI, 34.1–37.9%]) (Box 1, Box 2). In the youngest cohort, 12.3% (412 participants) experienced four to five maltreatment types and 25.4% (866 participants) experienced three to five types; this is comparable to the middle cohort, in which 13.4% (257 participants) experienced four to five types and 25.7% (502 participants) experienced three to five types. The rate of no maltreatment was highest for the oldest cohort (1173; 40.5% [95% CI, 38.5–42.4%]), lower for the youngest cohort (1368; 38.8% [95% CI, 37.0–40.6%]) and lowest for the middle cohort (682; 33.4% [95% CI, 31.2–35.7%]) (Box 1).

Box 2 Prevalence of single‐type maltreatment and multi‐type maltreatment (≥ 2 or ≥ 3 types), by age for men and women*

**Figure:**
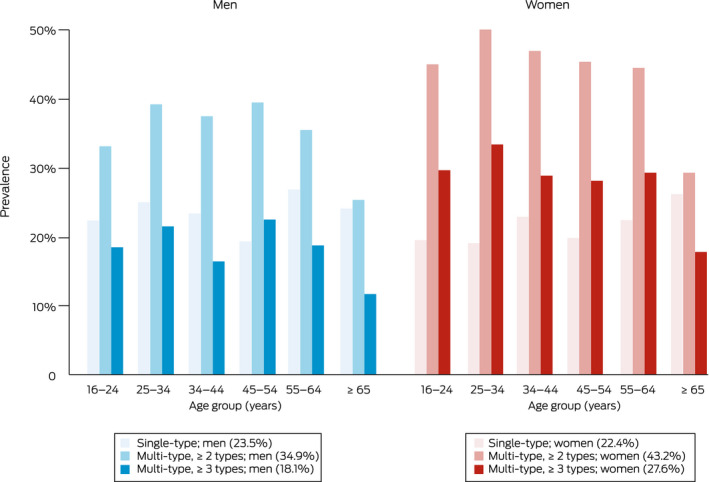
* Each percentage in parentheses is the proportion of the entire sample of men or women that falls into that category (eg, 23.5% of all surveyed men experienced single‐type maltreatment).

### Prevalence of different maltreatment domain combinations

To consider the nature of the experience of multi‐type child maltreatment, we examined all 26 possible combinations of the experienced maltreatment types (Box 3, Box 4; Supporting Information, tables 2–4). An estimated 719 500 Australians aged 16 years and older have experienced all five types of child maltreatment, representing 3.5% of the population (Box 3). Exposure to domestic violence occurred in all six of the most reported combinations (Box 4); in all possible combinations, it was experienced by an estimated 6 455 327 Australians (31.2%), but it was less frequently experienced alone (an estimated 1 727 300 Australians; 8.4%) (Supporting Information, table 1). Physical abuse and emotional abuse each featured in four of the six most reported combinations (all with ≥ 3% prevalence) (Box 4). Sexual abuse featured in three of the six most reported combinations, but neglect in only one (Box 4). Age‐group differences in the prevalence of multi‐type maltreatment were largely consistent with the overall trends when looking at each combination of child maltreatment types separately (Supporting Information, tables 2–4).^20^

Box 3 Prevalence of the five most common combinations of multi‐type maltreatment, and of single‐type maltreatment, showing the main patterns of overlap in multi‐type maltreatment combinations*

**Figure:**
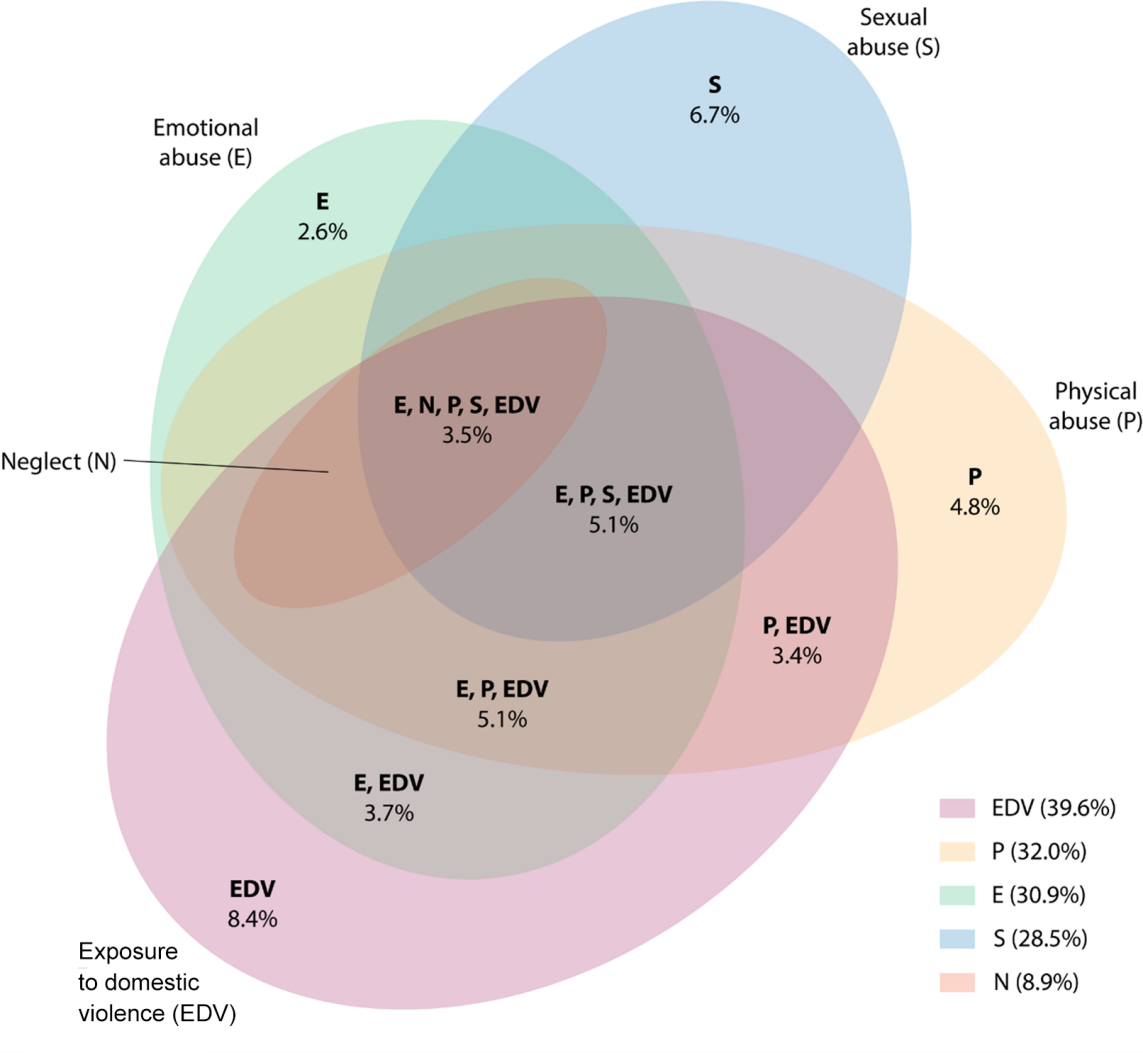
* Percentages at bottom right show overall prevalence of each maltreatment type.

Box 4 **The six most commonly reported combinations of multi‐type child maltreatment (≥ 3% prevalence)**

**Table:** 

Prevalence	Exposure to domestic violence	Emotional abuse	Physical abuse	Sexual abuse	Neglect
5.1%	✓	✓	✓	✓	
5.1%	✓	✓	✓		
3.7%	✓	✓			
3.5%	✓	✓	✓	✓	✓
3.4%	✓		✓		
3.0%	✓			✓

### Associations between family‐related risk factors and child maltreatment

For all four family‐related risk factors, there was a consistently increased risk of multi‐type (but not single‐type) maltreatment for participants with these risk factors compared with those without these risk factors (Supporting Information, tables 5–8). Overall, 16.4% of participants (1502) reported multi‐type maltreatment and parental separation or divorce (RR, 2.01 [95% CI, 1.89–2.14]) (Supporting Information, table 5); 16.1% (1535) reported multi‐type maltreatment and living with someone who was mentally ill, suicidal or severely depressed (RR, 2.42 [95% CI, 2.28–2.57]) (Supporting Information, table 6); 16.2% (1407) reported multi‐type maltreatment and living with someone who had a problem with alcohol or drugs (RR, 2.40 [95% CI, 2.26–2.55]) (Supporting Information, table 7); and 14.8% (1181) reported multi‐type maltreatment and family economic hardship (RR, 2.18 [95% CI, 2.06–2.32]) (Supporting Information, table 8). For each family‐related risk factor, presence of the risk factor was associated with more than double the risk of multi‐type maltreatment compared with absence of the risk factor.

Patterns were similar for women and men, and risks were even higher for participants with a diverse gender identity. Comparing age groups, the prevalence of experiencing family‐related risk factors and multi‐type maltreatment was highest for the middle cohort (25–44 years), compared with the youngest and oldest cohorts (16–24 and ≥ 45 years) (Supporting Information, tables 5–8).

## Discussion

To our knowledge, the ACMS is the first study globally to examine combined exposure to all five specific domains of child maltreatment in a representative sample. Elsewhere in this supplement, we report the prevalence rates for each type of maltreatment, ranging from 8.9% (neglect) to 39.6% (exposure to domestic violence).^20^ The current analysis of multi‐type maltreatment presents an important additional, and concerning, understanding of the experience of child maltreatment in Australia. Although more than one‐third of participants (37.8%) did not experience any type of child maltreatment, two in five (39.4%) experienced multi‐type maltreatment, nearly one‐quarter (23.3%) experienced three to five types, and more than one in ten (11.6%) experienced four to five types. Among participants aged 16–24 years, prevalence of any multi‐type maltreatment was slightly higher than for the whole sample, indicating that these experiences are not simply historical artefacts but reflect contemporary social trends that have major implications for public health policy and clinical practice.

Elsewhere in this supplement, we report that although women and men experience comparable rates of physical abuse and exposure to domestic violence, women experience higher rates of neglect, emotional abuse and, particularly, sexual abuse.^20^ We found similar trends for multi‐type maltreatment, with women being significantly more vulnerable than men (43.2% *v* 34.9%), and even higher vulnerability among Australians with diverse gender identities (66.1%). Across age groups, women were consistently more likely to have experienced multi‐type maltreatment. Although the youngest cohort of participants reported lower prevalence of physical abuse and specific subdomains of sexual abuse — suggesting that Australian society may have benefitted in recent decades from advances in policy, practice, social sensitisation, education and healthy parenting^20^ — this was not replicated in multi‐type maltreatment data for this cohort. This suggests exposure to multi‐type maltreatment may offset declines in individual maltreatment types. In addition, the high prevalence of multi‐type maltreatment in participants with a diverse gender identity (mostly in the youngest cohort) warrants specific focus on prevention and intervention strategies.

Across all possible multi‐type maltreatment combinations, those involving exposure to domestic violence were experienced by almost one in three Australians. This suggests the need for an important shift in the narrative around exposure to domestic violence, to consider it as a ubiquitous environmental pattern that is evident in almost a third of the population. Physical and emotional abuse also contributed strongly to these experiences, indicating a need for enhanced prevention of these maltreatment types, particularly in high risk families. In contrast, neglect seldom featured in the most common multi‐type maltreatment combinations. This contrasts with neglect being one of the more frequent harm types in children coming to the attention of statutory child protection authorities in Australia.^24^

Consistent with findings from studies conducted overseas,^16^ we found strong associations between multi‐type maltreatment and four family‐related risk factors. In descending order of risk, they were: living with someone who was mentally ill, suicidal or severely depressed; living with someone who had a problem with alcohol or drugs; experiencing family economic hardship; and parental separation or divorce. Our findings regarding multi‐type maltreatment risk align with those found in a 27‐year birth cohort study in Victoria^25^ — namely that economic disadvantage, poor parental mental health, parental substance misuse and social instability are associated with increased risk of maltreatment. Further analyses are required to determine whether known risk factors for child maltreatment can help differentiate between the occurrence of single‐type and multi‐type maltreatment, and to indicate suitable points of intervention.

Our findings reinforce the importance of statistically adjusting for multi‐type maltreatment to avoid overestimating the health and social effects when looking at associations with any single type of child maltreatment alone.^9^, ^10^, ^11^, ^12^, ^13^, ^14^ Understanding the overlap between different types of maltreatment changes our understanding of the nature of individual maltreatment types. Knowing that there is a high likelihood of each type being experienced in combination with other types, rather than in isolation, could affect the approaches to prevention and clinical intervention in response to an identified maltreatment type.

Our findings suggest that not only is the true prevalence of maltreatment far higher than the proportion of cases coming to the attention of government agencies, but that for the many Australians experiencing any form of child maltreatment (62.2%), the typical experience is of multi‐type maltreatment. Statutory child protection services and family support agencies need to consider the likelihood of multi‐type maltreatment. Equally, although we need to be careful about the expansion of screening without well tested tools, protocols and prepared interventions,^22^ health practitioners and mental health service providers should consider multi‐type maltreatment when engaged in clinical assessment and intervention, providing trauma‐informed therapeutic services that are designed to address the high likelihood that child maltreatment victims have been exposed to multi‐type maltreatment. Current public health prevention strategies need to move beyond singular maltreatment foci, and instead assess and manage the likelihood of experiencing multiple domains of child maltreatment, and the family‐related adverse childhood experiences such as poor parental mental health that increase the risk of multi‐type maltreatment.

Given that our prevalence estimates suggest that the experience of multi‐type maltreatment is almost twice as common as the experience of single‐type maltreatment (39.4% *v* 22.8%), prevention, protection and treatment services must coordinate interventions to respond to multi‐type maltreatment. Consideration should be given to the role of universal prevention programs and strategies aimed at a range of maltreatment types that children and adolescents experience, and the modifiable family‐related adversities that increase the likelihood of both single‐type and multi‐type maltreatment.

### Strengths and limitations

The ACMS captured nuanced representative population data about the experience of all five types of child maltreatment, enabling the identification of individuals most at risk — not only of each individual type of maltreatment, but also of multi‐type maltreatment in its different combinations. This knowledge is essential to develop evidence‐informed child protection policies, prevention strategies and interventions. Analysis of multi‐type maltreatment also enables assessment of its associations with mental health disorders, health risk behaviour and service use data, which are reported elsewhere in this supplement.^26^, ^27^, ^28^ Forthcoming analyses will examine differences in associated outcomes attributable to variability in age of onset, developmental periods of victimisation, chronicity, and particular multi‐type maltreatment combinations.

Cross‐sectional retrospective data do not allow ascertainment in all cases of the sequential timing and directionality of different types of maltreatment. Many children may experience multiple types of maltreatment in the same event, or at proximate times in childhood. However, the ACMS did capture data about age of onset and cessation for each type of maltreatment experienced. Although further analysis can examine the nature of developmental victimisation and its association with health and behavioural outcomes, we cannot be certain in all cases about the temporal progression of different maltreatment types. In addition, although major risk factors were examined in this analysis, not all possible family‐related risk factors were assessed. Further research should address the combined influence of multiple family‐related risk factors and other childhood adversities on the likelihood of experiencing multi‐type maltreatment and its associated outcomes.

### Conclusions

Multi‐type maltreatment is common and is the typical experience of Australians who experience any childhood maltreatment — it is almost twice as common as experiencing single‐type maltreatment. Compared with single‐type maltreatment, women are significantly more likely than men to have experienced multi‐type maltreatment, and people with a diverse gender identity are even more vulnerable. Exposure to domestic violence is the most prevalent individual maltreatment type, and features across the most frequent multi‐type maltreatment combinations. The relative risk of multi‐type maltreatment for individuals who have experienced other types of family‐based adversity (residing with someone with mental health problems or substance misuse problems, economic disadvantage, and parental separation or divorce) suggests that family supports could be an important prevention strategy for the most prevalent experience of child abuse and neglect: multi‐type maltreatment. Future studies with new youth samples could help establish whether the prevalence of multi‐type maltreatment is changing, and determine whether policy and prevention efforts to address risks of individual maltreatment types can be integrated to address risk of multi‐type maltreatment.

## Data access

The authors had full access to all the data (including statistical reports and tables).

## Data sharing statement

Under a registered data management plan, final datasets will be stored on the Australian Data Archive, with details for access from 2024 made available on the ACMS website (https://www.acms.au). Under a multi‐institutional agreement, the survey instrument is the intellectual property of the research team. It will be made available through a Creative Commons licence after an embargo period. For the purpose of open access, we have applied a Creative Commons Attribution (CC BY) license to any author‐accepted manuscript version arising from this submission.

## Open access

Open access publishing facilitated by Queensland University of Technology, as part of the Wiley ‐ Queensland University of Technology agreement via the Council of Australian University Librarians.

## Agency roles

The NHMRC funded the ACMS. The Australian Government and the Australian Institute of Criminology provided supplementary funding for several specific questions. The researchers were independent of the funders.

## Competing interests

No relevant disclosures.

## Supporting information


**Supporting Information**.
