# Supplementary material for: The prevalence and nature of multi‐type child maltreatment in Australia
Source: Med J Aust. 2023 Apr 2;218(Suppl 6):S19–25. doi: 10.5694/mja2.51868 (PMC10952595; doi:10.5694/mja2.51868)
Supplement: Supplementary file 1 — Supporting Information. [file MJA2-218-S19-s001.pdf]

Supporting Information

Supplementary results

This appendix was part of the submitted manuscript and has been peer reviewed.
It is posted as supplied by the authors.

Appendix to: Higgins DJ, Mathews B, Pacella R, et al. The prevalence and nature of multi-type child maltreatment in Australia. *Med J Aust* 2023; doi: 10.5694/mja2.51868.

Table 1. Single-type maltreatment only, by maltreatment type and gender*

	Participants — number; percentage (95% CI)					
	Emotional abuse	Neglect	Physical abuse	Sexual abuse	Exposure to domestic violence	Any single-type maltreatment
**All genders**	235; 2.6% (2.2–3.0%)	28; 0.4% (0.2–0.6%)	366; 4.8% (4.2–5.4%)	518; 6.7% (6.0–7.4%)	755; 8.4% (7.6–9.1%)	1902; 22.8% (21.7–24.0%)
Women	141; 3.1% (2.4–3.7%)	8; 0.1% (0.02–0.3%)	122; 3.4% (2.6–4.1%)	341; 8.7% (7.6–9.8%)	316; 7.1% (6.1–8.1%)	918; 22.4% (20.7–24.0%)
Men	93; 2.2% (1.6–2.8%)	20; 0.7% (0.3–1.0%)	234; 6.4% (5.5–7.4%)	169; 4.6% (3.7–5.4%)	429; 9.7% (8.6–10.8%)	954; 23.5% (21.8–25.1%)
Diverse genders	–	–	–	8; 5.5% (0.0–11.0%)	10; 9.1% (2.1–16.2%)	20; 15.4% (6.7–24.1%)

* Cells with *n* < 5 redacted.

Table 2. Multi-type maltreatment: any multi-type maltreatment (39.4% of whole sample) and two types of maltreatment (16.1% of whole sample)*

	Participants — percentage (95% CI)										
	**Any multi-type maltreatment**	**EA + Neg**	**EA + PA**	**EA + SA**	**EA + EDV**	**Neg + PA**	**Neg + SA**	**Neg + EDV**	**PA + SA**	**PA + EDV**	**SA + EDV**
**All ages**	39.4% (38.1–40.7%)	0.2% (0.1–0.4%)	2.0% (1.6–2.4%)	1.1% (0.8–1.3%)	3.7% (3.2–4.2%)	0.1% (0.0–0.2%)	0.2% (0.1–0.4%)	0.3% (0.2–0.5%)	2.0% (1.6–2.4%)	3.4% (2.9–3.9%)	3.0% (2.6–3.5%)
Women	43.2% (41.3–45.1%)	0.3% (0.1–0.5%)	1.9% (1.4–2.4%)	1.6% (1.1–2.1%)	3.3% (2.7–3.9%)	0.04% (0.0–0.1%)	0.4% (0.0–0.7%)	0.2% (0.0–0.3%)	2.0% (1.5–2.6%)	1.9% (1.4–2.5%)	3.9% (3.2–4.7%)
Men	34.9% (33.0–36.7%)	0.2% (0.0–0.3%)	2.2% (1.6–2.7%)	0.5% (0.2–0.7%)	4.1% (3.4–4.9%)	0.1% (0.0–0.2%)	–	0.5% (0.2–0.7%)	2.0% (1.4–2.5%)	5.0% (4.1–5.9%)	2.2% (1.6–2.7%)
Diverse genders	66.1% (53.7–78.7%)	–	–	2.6% (0.0–5.3%)	–	–	–	–	–	–	–
**16–24 years**	40.2% (38.4–42.0%)	0.2% (0.1–0.4%)	1.5% (1.0–1.9%)	1.3% (0.9–1.7%)	5.1% (4.3–5.9%)	–	0.2% (0.0–0.4%)	0.3% (0.1–0.4%)	1.1% (0.7–1.5%)	2.7% (2.2–3.3%)	2.4% (1.8–2.9%)
Women	45.6% (42.9–48.3%)	–	1.3% (0.7–1.9%)	2.3% (1.5–3.0%)	4.9% (3.8–6.1%)	–	–	0.3% (0.0–0.6%)	1.3% (0.8–1.9%)	1.5% (0.9–2.2%)	3.2% (2.3–4.2%)
Men	33.0% (30.6–35.5%)	0.3% (0.1–0.5%)	1.5% (1.0–2.1%)	–	5.4% (4.2–6.7%)	–	–	–	0.8% (0.3–1.4%)	4.0% (3.1–5.0%)	1.6% (0.9–2.3%)
**25–44 years**	44.0% (41.6–46.4%)	–	2.0% (1.3–2.7%)	1.0% (0.5–1.5%)	4.8% (3.7–5.8%)	–	–	0.5% (0.2–0.9%)	1.9% (1.2–2.6%)	3.9% (3.0–4.9%)	3.6% (2.7–4.5%)
Women	49.2% (45.7–52.6%)	–	2.0% (1.0–2.9%)	1.7% (0.7–2.6%)	4.2% (2.9–5.5%)	–	–	–	2.0% (1.0–3.0%)	2.1% (1.1–3.1%)	4.2% (2.8–5.6%)
Men	38.4% (35.0–41.7%)	–	2.0% (1.0–3.0%)	–	5.3% (3.7–7.0%)	–	–	0.6% (0.1–1.2%)	1.8% (0.9–2.7%)	5.9% (4.2–7.6%)	3.1% (1.9–4.3%)
**≥ 45 years**	36.0% (34.1–37.9%)	0.2% (0.0–0.4%)	2.2% (1.6–2.8%)	1.1% (0.7–1.4%)	2.6% (2.0–3.2%)	–	–	0.2% (0.0–0.3%)	2.3% (1.7–2.9%)	3.2% (2.5–3.9%)	2.8% (2.2–3.5%)
Women	38.7% (36.0–41.5%)	–	2.0% (1.2–2.8%)	1.4% (0.8–2.0%)	2.3% (1.6–3.1%)	–	–	–	2.2% (1.4–3.0%)	1.9% (1.2–2.7%)	4.0% (2.9–5.0%)
Men	32.8% (30.1–35.5%)	–	2.4% (1.6–3.3%)	0.7% (0.3–1.2%)	2.9% (2.0–3.8%)	–	–	–	2.4% (1.5–3.3%)	4.7% (3.4–5.9%)	1.6% (0.9–2.3%)

* Cells with $n < 5$ redacted. EA: emotional abuse; EDV: exposure to domestic violence; Neg: neglect; PA: physical abuse; SA: sexual abuse.

Table 3. Multi-type maltreatment: three types of maltreatment (11.7% of whole sample)*

	Participants — percentage (95% CI)									
	**PA + SA + EDV**	**Neg + SA + EDV**	**Neg + PA + EDV**	**Neg + PA + SA**	**EA + SA + EDV**	**EA + PA + EDV**	**EA + PA + SA**	**EA + Neg + EDV**	**EA + Neg + SA**	**EA + Neg + PA**
**All ages**	1.5% (1.2–1.8%)	0.2% (0.0–0.3%)	0.1% (0.0–0.2%)	0.1% (0.0–0.1%)	2.3% (1.9–2.7%)	5.1% (4.5–5.7%)	1.6% (1.2–1.9%)	0.5% (0.3–0.6%)	0.1% (0.0–0.2%)	0.3% (0.2–0.5%)
Women	1.5% (1.1–2.0%)	0.3% (0.1–0.5%)	–	–	3.3% (2.7–4.0%)	4.2% (3.4–4.9%)	2.1% (1.5–2.6%)	0.6% (0.3–0.9%)	0.2% (0.1–0.3%)	0.3% (0.1–0.5%)
Men	1.5% (1.0–2.0%)	–	0.2% (0.0–0.4%)	–	1.1% (0.8–1.5%)	6.0% (5.0–6.9%)	1.0% (0.6–1.4%)	0.3% (0.1–0.5%)	–	0.4% (0.1–0.6%)
Diverse genders	–	–	–	–	2.5% (0.4–4.6%)	9.0% (1.8–16.3%)	3.7% (0.0–8.2%)	–	–	–
**16–24 years**	1.3% (0.9–1.7%)	–	–	–	3.0% (2.3–3.6%)	6.0% (5.1–6.8%)	1.1% (0.7–1.4%)	1.0% (0.6–1.3%)	0.2% (0.1–0.4%)	0.3% (0.1–0.7%)
Women	1.6% (0.9–2.2%)	–	–	–	4.0% (3.0–5.0%)	5.3% (4.1–6.6%)	1.3% (0.7–1.9%)	1.0% (0.5–1.4%)	0.3% (0.0–0.6%)	–
Men	1.1% (0.6–1.6%)	–	–	–	1.8% (0.9–2.7%)	6.4% (5.2–7.7%)	0.7% (0.3–1.1%)	0.8% (0.4–1.3%)	–	0.5% (0.0–1.0%)
**25–44 years**	1.7% (1.1–2.3%)	–	–	–	2.1% (1.4–2.8%)	5.9% (4.7–7.0%)	1.4% (0.8–1.9%)	0.6% (0.2–0.9%)	–	–
Women	1.7% (0.9–2.6%)	–	–	–	3.3% (2.1–4.5%)	4.4% (2.9–5.8%)	2.1% (1.2–3.0%)	0.8% (0.2–1.4%)	–	–
Men	1.7% (0.8–2.5%)	–	–	–	0.9% (0.2–1.6%)	7.1% (5.3–9.0%)	0.5% (0.1–1.0%)	–	–	–
**≥ 45 years**	1.5% (1.0–1.9%)	0.2% (0.0–0.3%)	–	–	2.2% (1.6–2.8%)	4.3% (3.5–5.1%)	1.9% (1.3–2.4%)	0.2% (0.1–0.4%)	0.1% (0.0–0.3%)	0.4% (0.2–0.7%)
Women	1.4% (0.8–2.1%)	0.3% (0.0–0.6%)	–	–	3.2% (2.2–4.2%)	3.8% (2.7–4.9%)	2.3% (1.5–3.1%)	0.4% (0.1–0.7%)	0.2% (0.0–0.5%)	0.5% (0.1–0.9%)
Men	1.5% (0.8–2.2%)	–	–	–	1.1% (0.6–1.6%)	5.0% (3.7–6.2%)	1.4% (0.7–2.1%)	–	–	0.4% (0.0–0.7%)

* Cells with *n* < 5 redacted. EA: emotional abuse; EDV: exposure to domestic violence; Neg: neglect; PA: physical abuse; SA: sexual abuse.

Table 4. Multi-type maltreatment: four types of maltreatment (8.1% of whole sample) and all five types of maltreatment (3.5% of whole sample)*

	Participants — percentage (95% CI)					
	**Neg + PA + SA + EDV (all except EA)**	**EA + PA + SA + EDV (all except Neg)**	**EA + Neg + SA + EDV (all except PA)**	**EA + Neg + PA + EDV (all except SA)**	**EA + Neg + PA + SA (all except EDV)**	**EA + Neg + PA + SA + EDV (all five)**
**All ages**	0.1% (0.1–0.2%)	5.1% (4.6–5.7%)	0.6% (0.4–0.8%)	1.8% (1.4–2.2%)	0.4% (0.2–0.6%)	3.5% (3.0–4.0%)
Women	0.2% (0.1–0.4%)	6.7% (5.8–7.6%)	0.9% (0.5–1.3%)	1.8% (1.2–2.3%)	0.6% (0.3–1.0%)	4.7% (3.9–5.5%)
Men	–	3.3% (2.7–4.0%)	0.2% (0.0–0.4%)	1.9% (1.4–2.3%)	–	2.0% (1.5–2.6%)
Diverse genders	–	12.1% (4.4–19.9%)	3.0% (0.7–12.5%)	–	–	12.4% (13.6–21.2%)
**16–24 years**	0.3% (0.0–0.5%)	4.9% (4.1–5.7%)	1.2% (0.8–1.6%)	2.0% (1.5–2.5%)	0.3% (0.1–0.5%)	3.7% (3.0–4.4%)
Women	–	6.4% (5.1–7.7%)	1.7% (1.0–2.3%)	1.8% (1.1–2.4%)	0.4% (0.1–0.7%)	5.6% (4.4–6.8%)
Men	–	2.8% (2.0–3.7%)	0.5% (0.1–0.8%)	2.2% (1.5–2.9%)	–	1.4% (0.9–2.0%)
**25–44 years**	–	5.5% (4.5–6.6%)	0.5% (0.1–0.9%)	2.4% (1.6–3.1%)	0.4% (0.1–0.7%)	4.4% (3.4–5.5%)
Women	–	7.9% (6.0–9.7%)	0.8% (0.1–1.5%)	2.8% (1.6–3.9%)	–	6.2% (4.4–7.9%)
Men	–	3.2% (2.0–4.4%)	–	2.0% (1.1–3.0%)	–	2.4% (1.4–3.5%)
**≥ 45 years**	–	5.0% (4.1–5.8%)	0.5% (0.2–0.9%)	1.4% (0.9–1.8%)	0.4% (0.1–0.7%)	2.8% (2.1–3.4%)
Women	–	6.0% (4.8–7.3%)	0.8% (0.2–1.3%)	1.1% (0.5–1.7%)	0.8% (0.2–1.4%)	3.5% (2.5–4.5%)
Men	–	3.6% (2.6–4.6%)	–	1.6% (1.0–2.3%)	–	1.9% (1.1–2.7%)

* Cells with *n* < 5 redacted. EA: emotional abuse; EDV: exposure to domestic violence; Neg: neglect; PA: physical abuse; SA: sexual abuse.

Table 5. Parental divorce or separation and relative risk (RR) of no maltreatment, any single-type maltreatment or multi-type maltreatment

	No maltreatment		One maltreatment type only		Any multi-type maltreatment					
	n (%)	RR (95% CI)	*n* (%)	RR (95% CI)	*n* (%)	RR (95% CI)	Two types, *n* (%)	Three types, *n* (%)	Four types, *n* (%)	Five types, *n* (%)
**All ages**	467 (5.0%)	0.43 (0.38–0.48)	435 (4.8%)	0.75 (0.66–0.85)	1502 (16.4%)	2.01 (1.89–2.14)	474 (5.1%)	468 (5.0%)	374 (4.2%)	186 (2.1%)
Women	201 (4.2%)	0.38 (0.32–0.45)	199 (4.4%)	0.67 (0.56–0.81)	819 (18.0%)	1.98 (1,83–2.15)	233 (4.8%)	241 (5.2%)	219 (5.2%)	126 (2.9%)
Men	263 (5.9%)	0.49 (0.42–0.57)	227 (5.1%)	0.82 (0.69–0.97)	632 (14.4%)	2.05 (1.86–2.27)	233 (5.4%)	212 (4.8%)	135 (2.9%)	52 (1.2%)
Diverse genders	3 (3.4%)	0.24 (0.06–0.98)	9 (9.8%)	1.83 (0.64–5.26)	51 (34.1%)	1.24 (0.84–1.83)	8 (2.2%)	15 (10.2%)	20 (14.5%)	8 (7.2%)
**16–24 years**	263 (7.9%)	0.46 (0.40–0.52)	216 (6.2%)	0.76 (0.65–0.89)	748 (21.6%)	2.10 (1.92–2.29)	245 (7.0%)	233 (6.7%)	179 (5.4%)	91 (2.6%)
Women	116 (7.3%)	0.45 (0.37–0.55)	98 (6.0%)	0.72 (0.57–0.92)	398 (24.1%)	1.88 (1.68–2.11)	119 (7.0%)	121 (7.1%)	97 (6.3%)	61 (3.7%)
Men	146 (8.8%)	0.49 (0.42–0.58)	114 (6.5%)	0.82 (0.66–1.02)	310 (18.1%)	2.40 (2.08–2.78)	118 (6.9%)	101 (6.1%)	67 (3.8%)	24 (1.2%)
**25–44 years**	108 (5.9%)	0.41 (0.34–0.51)	119 (6.0%)	0.72 (0.58–0.88)	398 (21.9%)	1.94 (1.75–2.16)	130 (7.0%)	116 (6.4%)	100 (5.5%)	52 (3.0%)
Women	46 (4.8%)	0.34 (0.25–0.47)	55 (5.5%)	0.62 (0.46–0.84)	228 (25.9%)	1.96 (1.72–2.24)	69 (7.6%)	58 (6.4%)	65 (7.6%)	36 (4.4%)
Men	60 (6.8%)	0.50 (0.38–0.64)	61 (6.5%)	0.83 (0.62–1.10)	162 (17.6%)	1.89 (1.60–2.23)	61 (6.6%)	54 (6.1%)	33 (3.4%)	14 (1.5%)
**≥ 45 years**	96 (3.7%)	0.44 (0.36–0.54)	100 (3.6%)	0.79 (0.64–0.97)	356 (11.3%)	2.01 (1.81–2.22)	99 (3.2%)	119 (3.7%)	95 (3.0%)	43 (1.4%)
Women	39 (3.0%)	0.40 (0.29–0.55)	46 (3.3%)	0.74 (0.55–1.01)	193 (11.4%)	1.95 (1.71–2.23)	45 (2.4%)	62 (3.9%)	57 (3.4%)	29 (1.8%)
Men	57 (4.5%)	0.48 (0.37–0.62)	52 (3.7%)	0.80 (0.60–1.08)	160 (11.0%)	2.12 (1.81–2.47)	54 (4.2%)	57 (3.5%)	35 (2.3%)	14 (1.0%)

Table 6. Living with someone who was mentally ill, suicidal or severely depressed and relative risk (RR) of no maltreatment, any single-type maltreatment or multi-type maltreatment

	No maltreatment		One maltreatment type only		Any multi-type maltreatment					
	n (%)	RR (95% CI)	n (%)	RR (95% CI)	n (%)	RR (95% CI)	Two types, n (%)	Three types, n (%)	Four types, n (%)	Five types, n (%)
**All ages**	306 (2.9%)	0.29 (0.25–0.33)	336 (3.5%)	0.62 (0.54–0.71)	1535 (16.1%)	2.42 (2.28–2.57)	449 (4.7%)	451 (4.4%)	429 (4.7%)	206 (2.3%)
Women	147 (3.1%)	0.27 (0.22–0.34)	175 (3.4%)	0.49 (0.40–0.60)	946 (20.0%)	2.43 (2.24–2.63)	256 (5.2%)	266 (5.3%)	276 (6.2%)	148 (3.3%)
Men	154 (2.7%)	0.32 (0.26–0.39)	157 (3.6%)	0.84 (0.69–1.02)	532 (11.5%)	2.31 (2.09–2.54)	184 (4.1%)	169 (3.3%)	133 (3.0%)	46 (1.1%)
Diverse genders	5 (3.4%)	0.25 (0.08–0.85)	4 (2.1%)	0.18 (0.05–0.70)	57 (41.2%)	1.87 (1.24–2.82)	9 (4.9%)	16 (10.7%)	20 (13.7%)	12 (11.9%)
**16–24 years**	169 (4.5%)	0.31 (0.26–0.36)	164 (4.3%)	0.62 (0.52–0.74)	744 (20.9%)	2.57 (2.36–2.80)	219 (5.8%)	229 (6.3%)	198 (5.9%)	98 (2.9%)
Women	78 (4.6%)	0.28 (0.22–0.35)	92 (5.0%)	0.61 (0.48–0.78)	440 (26.1%)	2.42 (2.16–2.72)	120 (6.8%)	129 (7.4%)	120 (7.5%)	71 (4.4%)
Men	88 (4.5%)	0.39 (0.31–0.48)	69 (3.7%)	0.69 (0.51–0.89)	260 (14.2%)	2.62 (2.29–3.00)	91 (4.7%)	87 (4.8%)	63 (3.8%)	19 (1.0%)
**25–44 years**	75 (3.3%)	0.28 (0.22–0.36)	95 (4.6%)	0.65 (0.51–0.81)	400 (20.4%)	2.20 (1.99–2.43)	120 (6.2%)	103 (4.9%)	114 (5.9%)	63 (3.4%)
Women	36 (3.5%)	0.25 (0.18–0.36)	45 (4.5%)	0.51 (0.37–0.71)	257 (26.6%)	2.24 (1.96–2.56)	69 (7.1%)	65 (6.2%)	82 (8.6%)	41 (4.7%)
Men	38 (3.2%)	0.34 (0.24–0.48)	49 (4.7%)	0.89 (0.65–1.21)	133 (13.6%)	2.02 (1.71–2.37)	50 (5.3%)	35 (3.4%)	29 (3.1%)	19 (1.9%)
**≥ 45 years**	62 (2.2%)	0.29 (0.22–0.37)	77 (2.5%)	0.59 (0.46–0.75)	391 (11.9%)	2.54 (2.32–2.79)	110 (3.4%)	119 (3.5%)	117 (3.6%)	45 (1.5%)
Women	33 (2.5%)	0.30 (0.21–0.43)	38 (2.2%)	0.44 (0.31–0.62)	249 (14.3%)	2.54 (2.25–2.88)	67 (3.7%)	72 (4.1%)	74 (4.3%)	36 (2.2%)
Men	28 (1.9%)	0.27 (0.18–0.41)	39 (2.8%)	0.84 (0.6–1.17)	139 (9.3%)	2.49 (2.14–2.89)	43 (3.0%)	47 (2.9%)	41 (2.7%)	8 (0.5%)

Table 7. Living with someone who had a problem with alcohol or drugs and relative risk (RR) of no maltreatment, any single-type maltreatment or multi-type maltreatment

	No maltreatment		One maltreatment type only		Any multi-type maltreatment					
	n (%)	RR (95% CI)	*n* (%)	RR (95% CI)	*n* (%)	RR (95% CI)	Two types, *n* (%)	Three types, *n* (%)	Four types, *n* (%)	Five types, *n* (%)
**All ages**	199 (2.5%)	0.24 (0.21–0.29)	313 (3.9%)	0.70 (0.61–0.80)	1407 (16.2%)	2.40 (2.26–2.55)	406 (4.9%)	418 (4.7%)	389 (4.4%)	194 (2.3%)
Women	98 (2.7%)	0.25 (0.20–0.31)	163 (4.0%)	0.64 (0.53–0.78)	804 (18.8%)	2.25 (2.08–2.44)	208 (5.2%)	224 (4.9%)	239 (5.7%)	133 (3.0%)
Men	99 (2.3%)	0.25 (0.19–0.31)	147 (3.7%)	0.79 (0.64–0.96)	557 (13.2%)	2.57 (2.34–2.82)	192 (4.6%)	182 (4.4%)	132 (3.0%)	51 (1.3%)
Diverse genders	2 (3.1%)	0.31 (0.07–1.43)	3 (4.3%)	0.60 (0.15–2.39)	46 (31.7%)	1.44 (1.01–2.06)	6 (3.4%)	12 (7.8%)	18 (11.3%)	10 (9.2%)
**16–24 years**	72 (2.0%)	0.18 (0.14–0.23)	125 (3.5%)	0.69 (0.57–0.83)	605 (17.3%)	2.56 (2.37–2.77)	163 (4.4%)	179 (5.2%)	170 (5.1%)	93 (2.7%)
Women	34 (2.1%)	0.18 (0.13–0.26)	62 (3.6%)	0.61 (0.46–0.80)	332 (20.7%)	2.33 (2.10–2.58)	74 (4.4%)	95 (5.8%)	97 (6.3%)	66 (4.1%)
Men	38 (2.0%)	0.20 (0.15–0.28)	62 (3.7%)	0.86 (0.66–1.12)	237 (13.0%)	2.82 (2.48–3.21)	84 (4.4%)	74 (4.4%)	59 (3.2%)	20 (1.0%)
**25–44 years**	45 (2.2%)	0.20 (0.15–0.28)	94 (4.8%)	0.79 (0.63–0.99)	351 (18.5%)	2.13 (1.93–2.34)	107 (5.2%)	93 (5.0%)	97 (5.2%)	54 (3.1%)
Women	18 (2.0%)	0.17 (0.11–0.28)	52 (5.1%)	0.76 (0.56–1.04)	204 (22.4%)	2.00 (1.76–2.27)	52 (5.4%)	51 (5.5%)	67 (7.4%)	34 (4.1%)
Men	25 (2.2%)	0.23 (0.15–0.37)	42 (4.6%)	0.88 (0.64–1.22)	140 (14.2%)	2.22 (1.89–2.60)	54 (5.0%)	40 (4.4%)	28 (2.9%)	18 (1.9%)
**≥ 45 years**	82 (2.9%)	0.30 (0.24–0.37)	94 (3.3%)	0.64 (0.51–0.79)	451 (14.4%)	2.57 (2.34–2.82)	136 (4.7%)	146 (4.3%)	122 (3.8%)	47 (1.6%)
Women	46 (3.3%)	0.33 (0.24–0.44)	49 (3.4%)	0.58 (0.43–0.78)	268 (16.0%)	2.40 (2.11–2.72)	82 (5.2%)	78 (4.3%)	75 (4.4%)	33 (2.1%)
Men	36 (2.5%)	0.27 (0.19–0.38)	43 (3.1%)	0.69 (0.50–0.95)	180 (12.5%)	2.80 (2.43–3.23)	54 (4.3%)	68 (4.4%)	45 (3.0%)	13 (0.9%)

Table 8. Family economic hardship and relative risk (RR) of no maltreatment, any single-type maltreatment or multi-type maltreatment

	No maltreatment		One maltreatment type only		Any multi-type maltreatment					
	n (%)	RR (95% CI)	*n* (%)	RR (95% CI)	*n* (%)	RR (95% CI)	Two types, *n* (%)	Three types, *n* (%)	Four types, *n* (%)	Five types, *n* (%)
**All ages**	230 (3.0%)	0.31 (0.27–0.36)	258 (3.8%)	0.73 (0.63–0.85)	1181 (14.8%)	2.18 (2.06–2.32)	331 (4.4%)	327 (4.0%)	334 (4.0%)	189 (2.4%)
Women	98 (2.5%)	0.24 (0.19–0.31)	144 (3.9%)	0.68 (0.55–0.83)	706 (17.7%)	2.19 (2.02–2.37)	185 (4.9%)	196 (4.7%)	195 (4.9%)	130 (3.3%)
Men	130 (3.5%)	0.40 (0.33–0.49)	110 (3.7%)	0.82 (0.66–1.01)	436 (11.4%)	2.13 (1.93–2.36)	142 (4.0%)	120 (3.1%)	124 (3.0%)	50 (1.3%)
Diverse genders	2 (3.1%)	0.39 (0.08–1.77)	4 (3.1%)	0.48 (0.12–1.93)	39 (27.9%)	1.41 (1.01–1.98)	4 (1.6%)	11 (8.1%)	15 (10.3%)	9 (7.8%)
**16–24 years**	85 (2.5%)	0.31 (0.25–0.39)	82 (2.5%)	0.61 (0.48–0.77)	455 (13.0%)	2.18 (2.01–2.36)	119 (3.2%)	127 (3.6%)	131 (3.9%)	78 (2.4%)
Women	41 (2.5%)	0.28 (0.20–0.38)	50 (3.3%)	0.72 (0.54–0.99)	260 (16.0%)	1.93 (1.74–2.14)	62 (3.5%)	72 (4.4%)	72 (4.6%)	54 (3.5%)
Men	44 (2.6%)	0.42 (0.31–0.56)	29 (1.5%)	0.49 (0.34–0.71)	165 (8.9%)	2.44 (2.14–2.78)	53 (2.7%)	47 (2.6%)	48 (2.7%)	17 (0.9%)
**25–44 years**	59 (3.0%)	0.29 (0.22–0.38)	68 (3.9%)	0.61 (0.47–0.79)	347 (18.5%)	2.14 (1.94–2.37)	106 (5.7%)	84 (4.5%)	101 (5.2%)	56 (3.1%)
Women	26 (2.7%)	0.23 (0.15–0.35)	36 (4.2%)	0.58 (0.39–0.81)	219 (23.6%)	2.12 (1.86–2.41)	62 (6.6%)	54 (5.5%)	67 (7.2%)	36 (4.2%)
Men	31 (3.2%)	0.38 (0.27–0.55)	31 (3.5%)	0.69 (0.48–1.01)	120 (13.0%)	2.10 (1.79–2.47)	44 (4.9%)	27 (3.2%)	31 (3.1%)	18 (1.9%)
**≥ 45 years**	86 (3.1%)	0.33 (0.27–0.41)	108 (4.1%)	0.86 (0.71–1.06)	379 (12.7%)	2.19 (1.98–2.41)	106 (3.8%)	116 (3.8%)	102 (3.2%)	55 (1.9%)
Women	31 (2.4%)	0.26 (0.18–0.37)	58 (3.9%)	0.77 (0.58–1.03)	227 (14.4%)	2.26 (1.99–2.57)	61 (4.1%)	70 (4.3%)	56 (3.4%)	40 (2.6%)
Men	55 (3.9%)	0.41 (0.32–0.54)	50 (4.4%)	0.98 (0.74–1.31)	151 (10.9%)	2.08 (1.78–2.44)	45 (3.6%)	46 (3.2%)	45 (3.0%)	15 (1.1%)
